# Modified osteochondral autograft transplantation for steroid-induced osteonecrosis of femoral head in idiopathic thrombocytopenic purpura: a case report and literature

**DOI:** 10.1186/s12891-023-07108-z

**Published:** 2024-01-02

**Authors:** Yichen Gong, Zhaokai Jin, Haojin Zhou, Hai Su, Guoqian Chen, Ying Zhong, Peijian Tong

**Affiliations:** The First Affiliated Hospital of Zhejiang Chinese Medical University (Zhejiang Provincial Hospital of Chinese Medicine), Zhejiang Province, Hangzhou, 310006 China

**Keywords:** Femoral head, Osteonecrosis, Osteochondral autograft, Surgical hip dislocation

## Abstract

Osteochondral autograft transplantation (OAT) has been commonly applied in the knee and ankle while the technique has not yet been a popularity in the femoral head. In this article, we present a 28-year-old female patient, who has a history of 1-year-use of glucocorticoid in the treatment of idiopathic thrombocytopenic purpura, with steroid-induced osteonecrosis of the femoral head (SONFH). She underwent surgical hip dislocation, osteochondroplasty, OAT, and internal fixation. Her Harris Hip Score improved from 64 to 82 in 36 months to follow-up. The case is valuable considering that a single, instead of several, 1.5 cm autograft was harvested from the non-bearing part of the same femoral head. This modification dispensed with the need of surgery for harvesting autograft from knee or ankle and reduced the structural vulnerability brought by the multihole donor part of the femoral head.

## Background

Steroid-induced osteonecrosis of the femoral head (SONFH), characterized by decreased osteogenesis, angiogenesis, and increased adipogenesis, is bone death caused by using chronic glucocorticoids and most commonly affects the femoral head. The common symptoms are pain and a disability to bear weight. MRI is usually the best radiologic technique in the diagnosis of SONFH. It is difficult to identify SONFH, and the later stage of the disease is perceived irreversible. Both non-surgical and surgical therapies are employed in SONFH.

Total hip arthroplasty (THA) is the most efficient treatment of SONFH for the elderly. However, this option is not well accepted by young patients who demand better flexibility and endurance [1]. Mosaicplasty, which fills the necrotic area with a collage of multiple small plugs of cartilage collected from the healthy nonweight-bearing area, is a widely applied treatment option for full thickness cartilage lesions particularly in the knee and ankle, though few in the hip joint [2]. Currently, Burak et al. has utilized this technique in the preserving femoral head surgery of osteonecrosis of the femoral head caused by developmental hip dislocation (DDH). The outcome of this technique is favorable [3].

In this case report, we applied the surgical technique of OTA along with osteochondroplasty in a patient with SONFH who had serious hip pain and activity obstacle caused by osteochondral lesion of the femoral head. The necrotic area was successfully replaced with healthy cancellous bone and cartilage with surgical hip dislocation, osteochondroplasty, OAT, and internal fixation. The case is unique since the etiology was steroid, and a single 1.5 cm osteochondral graft was employed in OAT.

### Case presentation

A 28-year-old female was hospitalized with the complaints of pain and disability in her right hip for 10 months. She had one-year history of using methylprednisolone tablet in the treatment of idiopathic thrombocytopenic purpura in 2018. She did not consult doctor until she could not endure the tenderness brought by daily walking. On physical examination, the patient presented tenderness in the right groin, pain at limits of the abduction and internal rotation, and Patrick sign of right lower extremity was noted. The picture of anteroposterior position showed uneven signal in right femoral head. Computed tomography (CT) and magnetic resonance imaging (MRI) confirmed the osteochondral lesion at central and superior lateral part of femoral head. Thus, the patient was diagnosed as SONFH (ARCO II), and required repair for the cartilage and cancellous bone under it. The operation was designed to repair the weight-bearing area of the femoral head and seek to alleviate pain and disability without surgical involvement of other parts of the body. The operation was scheduled after the written informed consent was obtained from the patient.

**Fig. 1:**
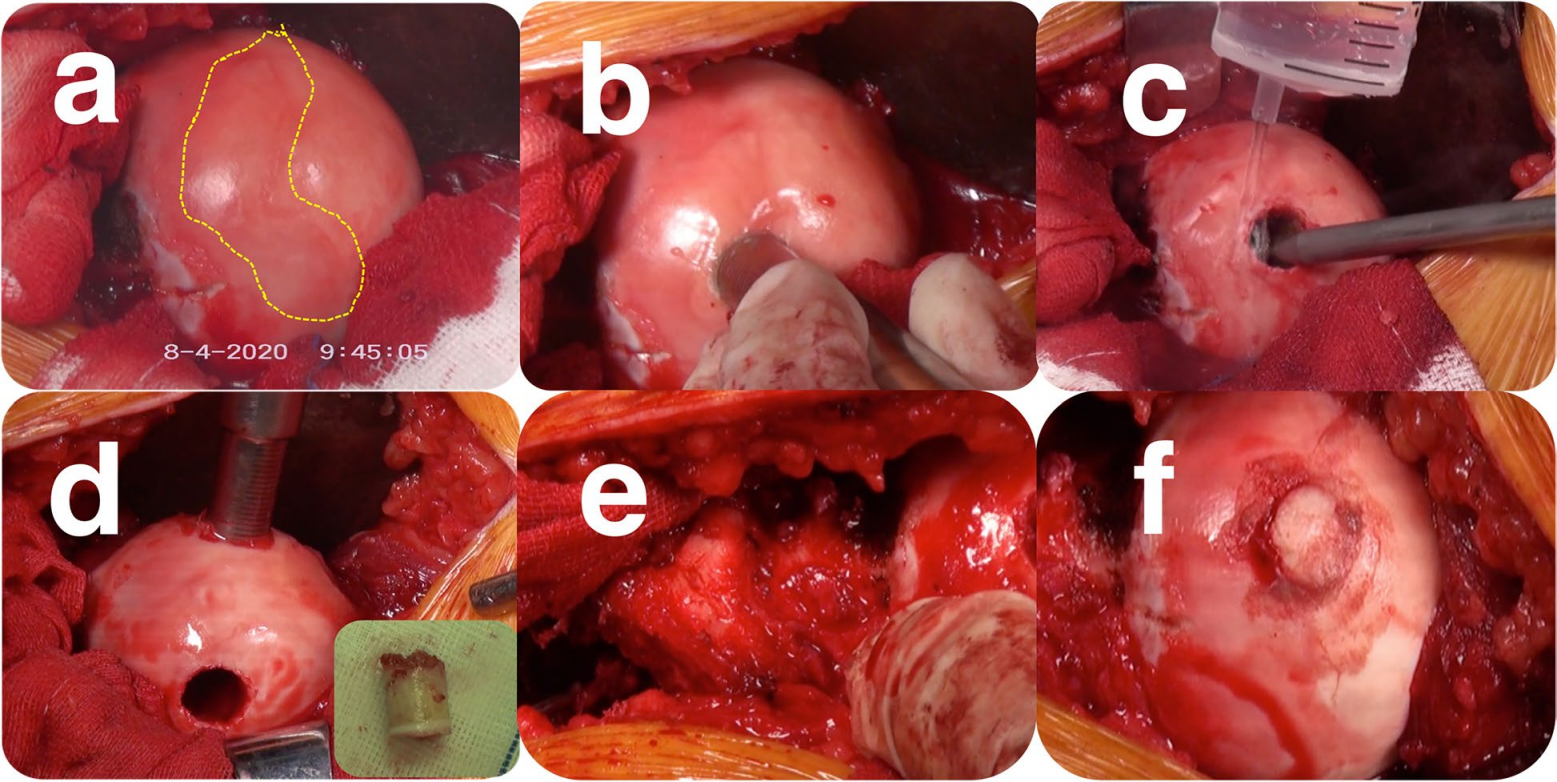
**a** The necrosis area. **b** a 1.5 × 1.5 cm area was debrided. **c**, irrigating the recipient site. **d** harvesting the autograft from the donor site. **e** harvesting the cancellous bone from the greater trochanter. **f** the completed OAT on femoral head

During the operation, general anesthesia was applied, and the patient was placed in the lateral decubitus position. A surgical dislocation of the right hip, according to the description of Ganz et al., was performed. A senior surgeon scrutinized the morphology of the femoral head and found the position of osteochondral lesion. A 1 × 4.5 cm lesion area was confirmed and a 1.5 × 1.5 cm area at the center was debrided. A 1.5 cm autograft was harvested from the inferior medial part of same femoral head. Both donor site and recipient site were filled with cancellous bone collected from the greater trochanter (Fig. 1). Then, OAT was performed on the femoral head with the autograft. At last, the trochanteric fragment was fixed with 4.5 mm cannulated and cortical screws.

**Fig. 2:**
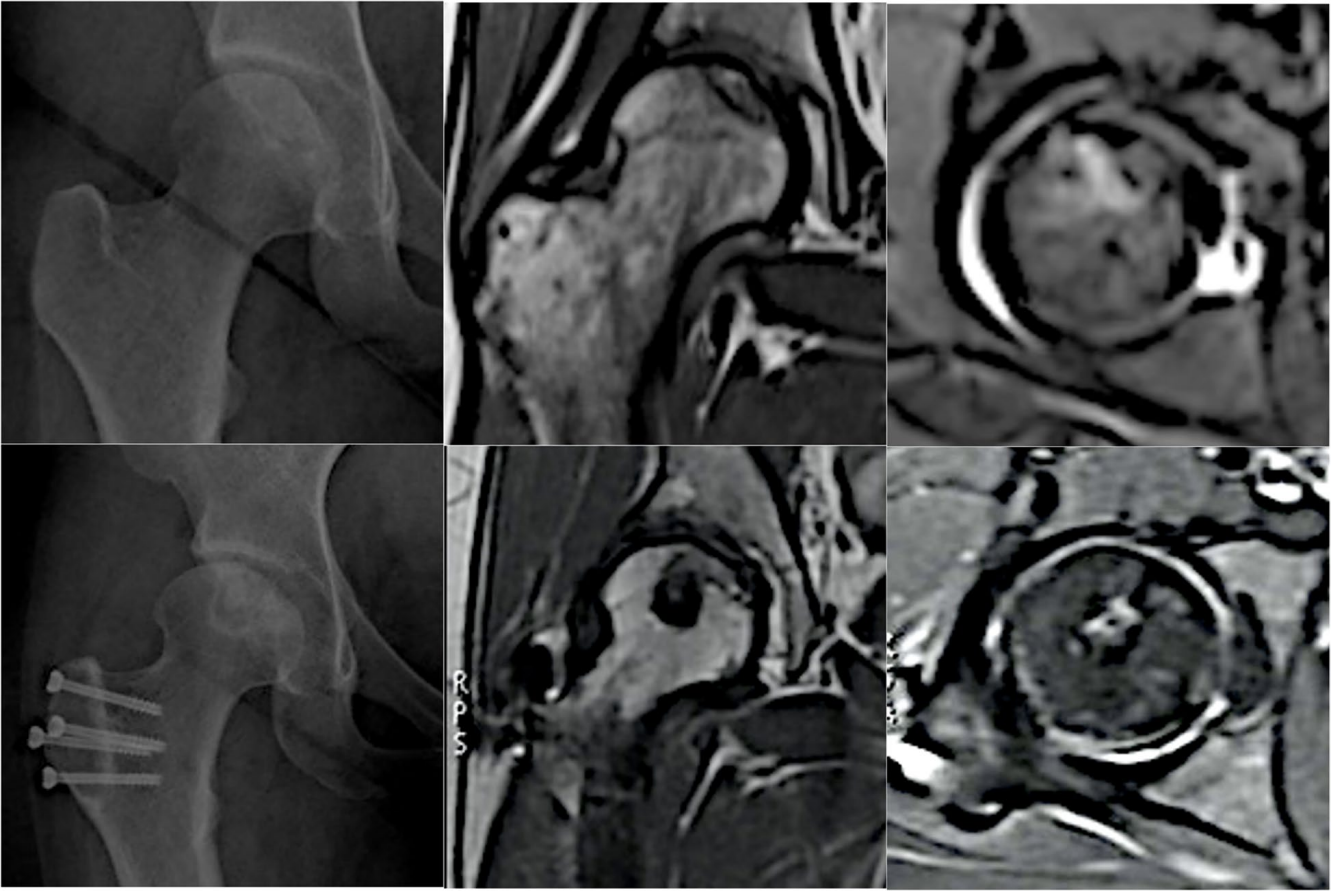
The anteroposterior images indicated the decent integration of the autograft and the femoral head had not collapsed. The magnetic resonance images showed the autograft and round shape of the femoral head

Functional exercise including continuous passive and quadriceps strengthening training was carried out by the patient in the postoperative period. The patient was allowed with a maximum 90° of hip flexion in the first 3 weeks, and abduction was prohibited for 4 weeks. Finally, she was asked to use crutches and carry out nonweight-bearing exercise for 3 months. In the third month, the patient had no complaint about movement limits or pain in daily life, and no complications were found. She was allowed to walk without crutches. After 9 months, anteroposterior images of the right hip showed the healing of trochanteric fragment and osteochondral integration. We encouraged her to come for a follow-up visit every year. 36 months later, MRI showed that the femoral head had not collapsed (ARCO II) and developed a cystic degeneration at the center of femoral head (Fig. 2). Her Harris Hip Score reached 82 and vas score was 0. The abduction of her operated hip reached 39° and the internal rotation reached 32°, and no tenderness was observed. Nevertheless, she could have sore hip after 1-h or longer sitting during which vas score could reach 2.

## Discussion and conclusions

In the present, the potential pathological process of SONFH remains unclear [3]. In surgical filed, THA is considered one of the most effective methods in the treatment of SONFH. Nevertheless, the rate of complications and reoperation is still high [4]. OAT, initially introduced by Hangodyand Füles [5] and gained its popularity in the knee and ankle, provides an approach of preserving femoral head surgery with the purpose of alleviating the pain and prolonging the service life of hip joint. In this case, we intended to preserve the morphology and function of the load-bearing area of femoral head by rebuilding the blood supply and preventing the development of the disease in this area. Therefore, the lesion of the load-bearing area was debrided and replaced by a healthy autograft from non-bearing part of the femoral head. According to the MRI and X-ray images, the lesion of non-bearing area further developed, while the load-bearing part remained integrated and round shape. Although the outcome of this surgery was favorable, follow-up visit every year was recommended as the development of SONFH had not stopped and the chance of reoperation remained high.

This case should be considered valuable for several reasons. First, this is the first OAT performed in SONFH that the donor site and recipient site are both in femoral head but not two different surgical areas, which reduces post-operation recovery time, surgical time and surgical bleeding. Second, combined with surgical hip dislocation technique, the surgeons can debride the necrosis area under direct vision, thus the arterial supply to the femoral head can be well preserved. Besides, different from mosaicplasty, this study utilized a single thicker osteochondral autograft, which provided stronger support for the femoral head and reduced the wound of donor site. Finally, compared with transtrochanteric rotational osteotomy that aims to fix the necrosis of wight-bearing area by rotating the proximal femur along the longitudinal axis of femoral shaft, this modified OAT has lower complexity and small surgical wound.

There are a few limits of this case. First, the cancellous bone collected from the greater trochanter failed to rebuild the recipient site, which developed a cystic degeneration in femoral head and increased the risk of collapse. Second, the follow-up time was only 36 months. Longer follow-up period is required to fully determine the long-term safety and efficacy of this OAT surgical technique.

Currently, although OAT has been mainly applied in the knee and ankle, there are limited studies that reported OAT in femoral head. Güngör et al. [6] utilized OAT along with osteochondroplasty of the femoral head in two patients with femoroacetabular impingement. Kilicoglu et al. [7], Gagala et al. [8], Hart et al. [9] harvested autograft from knee during OAT. Kubo et al. [10] used arthroscopic instead of surgical hip location as the surgical technique in the treatment of osteochondritis. Yoshihisa Tanaka et al. [11] applied a femoral autograft in osteonecrosis of the knee. In a case of anterior hip dislocation, Hyeonjoon Lee et al. [12] repaired the cartilage defect with multiple osteochondral plugs that obtained from the nonweight-bearing portion of the femoral head. Other etiologies in literature include trauma sequelae, sequelae of avascular necrosis, Legg-Calve-Perthes disease, multiple epiphyseal dysplasia and DDH. Considering the above, this case is unique because this is the first case reported using OAT, instead of Mosaicplasty, along with osteochondroplasty in the treatment of SONFH, and the autograft was collected from the nonweight-bearing portion of femoral head, which avoided unnecessary knee surgery (Table 1).

**Table 1:** Literature review of osteochondral autograft transplantation to femoral head

Author	Age/Gender	Etiology	Surgical tec	Follow-up	Defect size	Harvest site	Preoperative HSS	Postoperative HSS
Güngör et al. [7]	22/F 23/	Impingement Impingement	SHD SHD	14 months 12months	2.7 cm^2^ 3.6 cm^2^	Knee Knee	52 46	93 85
Burak et al. [3]	15/F	DDH	SHD	18 months	NR	Nonweight-bearing portion of femoral head	55	90
Kilicoglu et al. [8]	27/M	Sequelae of AVN	SHD	8 years	NR	Knee	55	96
Gagala et al. [9]	34/M 20/M 43/M	Trauma sequelae Trauma sequelae Trauma sequelae	SHD SHD SHD	80 months 62 months 24 months	NR NR NR	Knee Knee Knee	NR NR NR	98 100 90
Hart et al. [10]	28/M	Trauma sequelae	SHD	6 months	14 mm^2^	Knee	69	100
D'Lima et al. [13]	15/M 21/M	Trauma sequelae Trauma sequelae	SHD SHD	12 months 5 years	20×5 mm 10 mm^2^	Knee Nonweight-bearing portion of femoral head	NR NR	NR NR
Won et asl. [14]	31/M	Trauma sequelae	SHD	12 months	2.5×1 cm	Knee	NR	82
Anthonissen et al. [15]	20/M	Trauma sequelae	SHD	2 years	2×2.5 cm	Knee	NR	82
Lee et al. [16]	62/M	Trauma sequelae	SHD	2 years	2×2.3 cm	Nonweight-bearing portion of femoral head	NR	NR
Soteranos et al. [17]	32/M	Osteonecrosis	SHD	66 months	NR	Nonweight-bearing portion of femoral head	45	96
Kubo et al. [18]	37/F	OCD	SHD	NR	8.5 mm^2^	Knee	NR	NR
Louahem et al. [19]	15/F 16/F	OCD OCD	SHD SHD	4 years 18 months	2 cm^2^	Nonweight-bearing portion of femoral head Knee	52	93
Emre et al. [20]	22/M	LCPD	SHD	3 years	1.5 cm^2^ 8×18 cm	Knee Knee	46 43	85 96
Girard et al. [21]	10 cases Mean age: 18 (15-21)	6 LCPD, 4 MED	SHD	Mean 29.2 months (20-39)	4.8 cm^2^ (3-9)	Nonweight-bearing portion of femoral head	52.8 (35-74)	79.5 (65-93)

In conclusion, this case showed relative effectiveness of the employment of OAT in the treatment of SONFH by alleviating the pain, improving the joint movement, and extending the service life of hip joint. For those who are refused to receive THA and younger people, this modified OAT is an option in hip preservative surgery, although further studies are required.

## Data Availability

The datasets used and/or analyzed during the current study are available from the corresponding author on reasonable request.

## Abbreviations

OAT: Osteochondral autograft transplantation
SONFH: Steroid-induced osteonecrosis of the femoral head
THA: Total hip arthroplasty
DDH: Developmental hip dislocation
MRI: Computed tomography (CT); magnetic resonance imaging
